# Severe obesity associates with maladaptive glomerular haemodynamics clustered with insulin resistance and endothelial dysfunction

**DOI:** 10.1093/ckj/sfaf334

**Published:** 2025-11-03

**Authors:** Diego Moriconi, Chiara Rovera, Francesco Raggi, Silvia Armenia, Rosa Maria Bruno, Anna Solini

**Affiliations:** Department of Clinical and Experimental Medicine, University of Pisa, Pisa, Italy; Department of Information Engineering, University of Pisa, Pisa, Italy; Department of Clinical and Experimental Medicine, University of Pisa, Pisa, Italy; Department of Surgical, Medical, Molecular and Critical Area Pathology, University of Pisa, Pisa, Italy; Department of Clinical and Experimental Medicine, University of Pisa, Pisa, Italy; INSERM U970 Team 7, Paris Cardiovascular Research Centre – PARCC, Université Paris-Cité, Paris, France; Department of Surgical, Medical, Molecular and Critical Area Pathology, University of Pisa, Pisa, Italy

**Keywords:** arterial stiffness, endothelial dysfunction, glomerular filtration rate, insulin sensitivity, obesity

## Abstract

**Background:**

Obesity is a leading risk factor for chronic kidney disease, with glomerular hyperfiltration as one of its earliest manifestations. However, absolute glomerular filtration rate (GFR) does not distinguish well between a pressure-driven hyperfiltration and the physiological forms. Insulin resistance and endothelial dysfunction have been proposed as key correlates of maladaptive renal haemodynamics, but their interplay remains unclear.

**Methods:**

Cross-sectional pilot study involving 27 adults with severe obesity (mean body mass index of 43.9 kg/m²). Measured GFR (mGFR) was assessed by iohexol plasma clearance and effective renal plasma flow (ERPF) by ^123^I-ortho-iodohippurate clearance. A hyperfiltration index (P-score) was derived as the standardized difference between filtration fraction and effective renal plasma flow with higher values reflecting a maladaptive phenotype. Endothelial function was assessed by brachial artery flow-mediated dilation and insulin sensitivity by oral glucose insulin sensitivity.

**Results:**

Participants were stratified into tertiles of P-score. Across tertiles, mGFR did not differ, but ERPF declined (756 ± 113 vs 505 ± 130 mL/min, *P* = .001) and of filtration fraction increased (16 ± 3% vs 26 ± 5%, *P* = .001) from the lowest to the highest tertile. Insulin sensitivity significantly decreased with higher P-score (384 ± 50 vs 308 ± 33, *P* = .019). Endothelial function was reduced in the highest vs lowest tertile (3.44 ± 1.49 vs 6.21 ± 1.76%, *P* = .014), while responses to nitroglycerin did not differ. In univariate analyses, P-score was inversely associated with insulin sensitivity (r = –0.36, *P* = .036) and endothelial dysfunction (r = –0.40, *P* = .022), whereas measured mGFR showed no associations. In multivariable models, the link between P-score and insulin sensitivity remained significant after adjustment.

**Conclusion:**

A maladaptive phenotype characterized by elevated filtration fraction relative to renal plasma flow clustered with insulin resistance and endothelial dysfunction, whereas mGFR alone failed to capture these relationships. Comprehensive haemodynamic profiling may help to refine risk stratification in obesity.

KEY LEARNING POINTS **What was known:**

- Hyperfiltration in obesity may be pressure-driven, with disproportionate elevation of filtration fraction relative to renal plasma flow.

**This study adds:**

- Insulin resistance, not body mass index, drives pressure-driven hyperfiltration and endothelial dysfunction.

**Potential impact:**

- Glomerular filtration rate (GFR) alone does not capture these patterns, whereas combining GFR with ERPF refines kidney phenotyping.

## INTRODUCTION

Obesity is a global health challenge and a risk factor for chronic kidney disease. Glomerular hyperfiltration, defined as abnormally high glomerular filtration rate (GFR) [1], is one of the earliest renal alterations in obesity. Once considered an adaptive response to greater metabolic demand, persistent hyperfiltration imposes mechanical load on glomeruli, leading to podocyte injury, albuminuria and nephron loss [2, 3].

Growing evidence shows that hyperfiltration is not uniform, and multiple mechanisms may underlie the abnormally high GFR in obesity. Hyperfiltration can reflect a disproportionate rise in filtration fraction (FF), consistent with increased intraglomerular pressure [4, 5]. This resembles early diabetic nephropathy, where pressure overload accelerates podocyte injury and glomerulosclerosis [6, 7]. In other conditions, hyperfiltration stems from increased renal plasma flow with normal filtration fraction, reflecting a physiological adjustment whereby higher perfusion supplies the greater metabolic demand, as seen in pregnancy [1, 8]. Importantly, these distinctions are blunted when hyperfiltration is defined only by absolute or indexed GFR, which cannot separate pressure overload from increased perfusion. Even when measured, GFR alone does not clarify the mechanism or its prognostic relevance without data on plasma flow and FF.

A growing body of research implicates insulin resistance as a central driver of hyperfiltration in obesity. Hyperinsulinemia alters renal haemodynamics, enhances proximal tubular sodium reabsorption and increases glomerular pressure [9, 10]. Consistently, human studies show that after adjusting for insulin sensitivity the link between adiposity and hyperfiltration is markedly attenuated [11, 12] and that insulin-resistant individuals exhibit higher GFR than insulin-sensitive counterparts despite comparable body mass index (BMI) [13].

Furthermore, obesity is consistently associated with endothelial dysfunction. Insulin resistance reduces nitric oxide bioavailability and impairs endothelium-dependent vasodilation [14], and obese insulin-resistant individuals show worse endothelial function than insulin-sensitive counterparts independent of BMI [15, 16], a process potentially aggravated by hyperfiltration-induced shear stress [14].

Taken together, insulin resistance, hyperfiltration and endothelial dysfunction represent a pathophysiological triad that amplifies cardio-renal risk in obesity. Clarifying their interplay may improve the characterization of the obese kidney phenotype.

The aim of this study was to evaluate whether a haemodynamic index of hyperfiltration could identify a maladaptive renal phenotype in severe obesity and to explore its links with metabolic and vascular alterations. Specifically, we examined whether such a maladaptive hyperfiltration pattern was associated with greater endothelial dysfunction and higher degrees of insulin resistance.

## MATERIALS AND METHODS

This cross-sectional study enrolled 30 consecutive adult subjects with obesity referring to the Outpatient Clinic for Metabolic Diseases of the University Hospital of Pisa between 2018 and 2019.

Three participants were excluded: two due to non-interpretable iohexol/p-aminohippuric acid curves from intravenous extravasation and one for incomplete oral glucose tolerance test (OGTT) with missing oral glucose insulin sensitivity index (OGIS). The final analytic sample comprised 27 participants.

The study protocol was approved by the local Ethics Committee (n. 3463/2011 and subsequent amendments), and all participants provided a written informed consent.

Exclusion criteria were an estimated GFR (eGFR) <60 mL/min/1.73 m², calculated by the Chronic Kidney Disease Epidemiology Collaboration (CKD-EPI) equation and the presence of albuminuria >30 mg/g, which was excluded by nephelometric analysis at the screening visit, systemic inflammatory diseases, cancer or heart/liver failure. Known diabetes was an exclusion criterion. All participants underwent a baseline OGTT; however, those meeting American Diabetes Association (ADA) thresholds were classified as newly diagnosed diabetes and retained in the main analyses. OGIS was the primary metabolic exposure. Angiotensin-converting enzym inhibitors/angiotensin-receptor blockers were discontinued 1 week before renal haemodynamic testing, while calcium-channel blockers were permitted for blood pressure control, as they are not known to directly affect glomerular haemodynamic.

### Experimental session

Participants underwent detailed history, physical examination, office blood pressure measurement after 10 min supine rest (OMRON-705IT, Kyoto, Japan) and fasting venous sampling for standard biochemistry.

### Dietary assessment

All participants were instructed to complete a 3-day food diary (two weekdays and one weekend day) within 2 weeks of the haemodynamic study. Diaries were reviewed by a trained dietitian. For each subject, mean daily energy intake and macronutrient composition were calculated. Protein intake was additionally expressed as g/kg/day using adjusted body weight, calculated as: ideal body weight (IBW) + 0.25 × (actual body weight – IBW), where IBW was derived from the Devine formula [17].

### Oral glucose tolerance test

After an overnight fast (12 h), all subjects consumed an oral glucose load consisting of 150 mL of 50% dextrose solution. Blood samples were collected through an indwelling cannula at times −15, 0, 15, 30, 60, 90, 120, 180 min during the test to measure plasma glucose and insulin. Plasma glucose was measured immediately by the glucose-oxidase technique (Beckman Glucose Analyzer II, Fullerton, CA, USA). Blood samples were centrifuged for 15 min (3000 × *g* at 4°C) and frozen at −20°C before analysis. Insulin measurements were performed by electrochemiluminescence on a COBAS e411 instrument (Roche, Indianapolis, IN, USA). Insulin sensitivity was assessed using the OGIS [18]. OGIS has been validated against the euglycemic–hyperinsulinemic clamp and provides a dynamic estimate of whole-body insulin sensitivity, particularly reflecting peripheral glucose disposal [19] (Supplementary methods).

### Arterial tonometry

Aortic stiffness was assessed by carotid–femoral pulse wave velocity (cf-PWV) using applanation tonometry (SphygmoCor, AtCor Medical, Sydney, Australia), following current guidelines. Central haemodynamics and augmentation index (Aix@75) were derived using the manufacturer’s validated transfer function, with brachial pressure calibration as previously described [20]. Full methodological details are provided in the Supplementary methods.

### Endothelial function

Methods for the assessment of flow-mediated dilation (FMD) of the brachial artery, including both traditional and allometric approaches, have been previously described [20] and assessments were conducted in accordance with internationally recognized expert consensus protocols. Briefly, a cuff was placed around the right forearm, and the right brachial artery was identified and scanned longitudinally 5–10 cm above the elbow using a 10-MHz linear array transducer (MyLab 25; Esaote, Florence, Italy), stabilized by a stereotactic clamp. The cuff was inflated to 300 ± 30 mmHg and deflated after 5 min. Brachial artery diameter and flow velocity were continuously measured using a real-time computerized edge detection system (Cardiovascular Suite; Quipu srl, Pisa, Italy) for 1 min at baseline, during cuff inflation, and for 4 min following cuff deflation. Endothelium-independent vasodilation was assessed after the sublingual administration of 25 µg of glyceryl trinitrate (GTN).

### Renal functional measurements

GFR was assessed by plasma clearance of iohexol, and effective renal plasma flow (ERPF) by plasma clearance of ^123^I-orthoiodohippurate as previously described [21], using validated multisample protocols [22] (see Supplementary methods). FF was calculated as GFR/ERPF, and renal vascular resistance (RVR) as mean arterial pressure divided by renal blood flow. GFR and ERPF were expressed both as absolute values and indexed for body surface area and height.

### Hyperfiltration index (P-score)

To operationalize the distinction between maladaptive (pressure-mediated) and adaptive hyperfiltration in obesity, we derived a continuous P-score based on the standardization of FF and ERPF within the study cohort. The P-score was calculated as follows:

FF = mGFR/ERPF; standardized FF and ERPF across the cohort to obtain their z-scores:

z(FF) = (FF_i_ – μFF)/σFF; z(ERPF) = (ERPF_i_ – μERPF)/σERPF

Define the P-score_i_ = z(FF_i_) – z(ERPF_i_)

A higher P-score thus indicates a disproportionately elevated FF relative to renal plasma flow, reflecting a maladaptive haemodynamic pattern characterized by increased glomerular pressure. Conversely, lower or negative P-score values suggest hyperfiltration primarily driven by high renal perfusion, reflecting a more physiologic haemodynamic state. For descriptive figures, the P-score was rescaled to a 0–1 range using min-max normalization, though all statistical models employed the original z-difference formulation.

### Statistical analysis

Continuous variables were expressed as mean ± standard deviation or median (interquartile range), according to distribution (Shapiro–Wilk test). Categorical variables were given as counts and percentages. Between-group comparisons used *t*-test or Mann–Whitney U for continuous data and Chi-square or Fisher’s exact test for categorical data. FMD was analysed with an allometric scaling approach, using ln(peak – baseline diameter) as outcome and ln(baseline diameter) as covariate. Associations between continuous variables were explored by Pearson or Spearman correlation coefficients, according to distribution. To further examine the relationships of interest, multivariable linear regression models were constructed. Specifically, OGIS and FMD were modelled separately as dependent variables, with P-score as the main predictor, adjusting for potential confounders in distinct models to limit overfitting. Robustness of the P-score–insulin sensitivity association was tested by a two-sided permutation procedure, with empirical *P*-values derived from the proportion of permuted |r| as extreme as observed.

As sensitivity analysis, all primary associations were reassessed after excluding participants who met ADA diagnostic criteria for diabetes at baseline OGTT.

Statistical tests were performed using JMP Pro 17.3.0 (SAS Institute Inc., Cary, NC, USA) using a two-sided α level of 0.05.

## RESULTS

The cohort comprised 27 individuals with severe obesity (age 46 ± 12 years, 70% women, BMI 43.9 ± 7.2 kg/m²); 19% were current smokers, 30% had well controlled hypertension, and 39% dyslipidaemia. Four participants were newly diagnosed with diabetes at baseline OGTT. Kidney function was preserved, with mGFR 121 ± 20 mL/min and eGFR 99 ± 16 mL/min/1.73 m². Full baseline characteristics are reported in Supplementary data, Table S1. Study participants were then stratified into tertiles according to the distribution of the P-score, ranging from the third tertile with the highest P-score (representing the most maladaptive pattern) to the first tertile with lowest P-score.

### Clinical and metabolic characteristics

Across P-score tertiles, age, sex distribution, BMI, blood pressure, lipid profile and HbA1c did not significantly differ. In contrast, insulin sensitivity, as estimated by OGIS, progressively declined with increasing P-score values, being significantly lower in the highest tertile compared with the lowest (308 ± 33 vs 384 ± 50 mL/min*m⁻², *P* = .019). High-sensitivity C-reactive protein (hs-CRP) levels did not differ significantly between P-score groups (Table 1).

**Table 1:** Clinical and laboratory characteristics of the study population stratified by P-score tertiles.

	First tertile	Second tertile	Third tertile	
	(low P-score)	(middle P-score)	(high P-score)	*P*-value
*N*	9	9	9	
P-score	–1.27 (–2.68 to –1.09)	–0.17 (–0.32 to 0.01)	1.49 (0.68–3.03)	**<.001**
Age (years)	42 ± 12	44 ± 13	52 ± 9	.139
Sex, female, *n* (%)	7 (78)	7 (78)	5 (56)	.491
Weight (kg)	128 ± 25	117 ± 16	119 ± 22	.561
BSA (m^2^)	2.27 ± 0.23	2.21 ± 0.16	2.25 ± 0.29	.899
BMI (kg/m^2^)	46.8 ± 9.2	42.3 ± 6.5	42.0 ± 4.7	.408
Smoking, *n* (%)	1 (11)	2 (22)	2 (22)	.782
Dyslipidemia, *n* (%)	3 (33)	3 (33)	4 (33)	.853
Hypertension, *n* (%)	2 (22)	2 (22)	4 (44)	.491
Type 2 diabetes, *n* (%)	1 (11)	1 (11)	2 (22)	.745
SBP (mmHg)	126 ± 11	125 ± 14	129 ± 16	.824
DBP (mmHg)	77 ± 9	78 ± 10	81 ± 10	.766
Heart rate (bpm)	74 ± 11	69 ± 11	68 ± 8	.373
eGFR (mL/min/1.73 m^2^)^a^	105 ± 21	99 ± 15	92 ± 13	.206
eGFR (mL/min)	136 ± 27	127 ± 25	122 ± 24	.557
Glycaemia (mg/dL)	100 ± 26	95 ± 19	107 ± 16	.292
HbA1c (%)	6.0 ± 0.6	6.1 ± 0.5	6.3 ± 0.8	.444
OGIS (mL/min*m^−2^)^b^	384 ± 50	363 ± 72	308 ± 33*	**.019**
Total cholesterol (mg/dL)	185 ± 18	178 ± 28	188 ± 27	.699
HDL (mg/dL)	40 ± 12	45 ± 10	42 ± 12	.578
LDL (mg/dL)	119 ± 18	118 ± 20	121 ± 29	.198
Triglycerides (mg/dL)	131 (85–150)	96 (82–184)	116 (95–125)	.871
hs-CRP (mg/L)	3.0 ± 1.4	2.7 ± 1.3	2.8 ± 1.5	.433

Values are mean ± standard deviation or median (interquartile range) unless otherwise specified.

^a^eGFR by CKD-EPI 2021 equation.

^b^OGIS, 75 g OGTT.

Comparisons across tertiles were performed using analysis of variance or Kruskal–Wallis test for continuous variables and Chi-square test for categorical variables.

*P < .05 vs first tertile (low P-score).

BSA, body surface area; DBP, diastolic blood pressure; SBP, systolic blood pressure; HDL, high-density lipoprotein; LDL, low-density lipoprotein.

Bold values indicate *P* < .05

### Dietary regimen

Mean daily macronutrient composition was comparable between groups. In patients with high P-score, proteins accounted for 17.8 ± 3.9% of total energy intake, carbohydrates for 57.1 ± 8.2% and lipids for 24.2 ± 3.8%. In patients with low P-score, proteins accounted for 17.2 ± 4.0%, carbohydrates for 55.2 ± 7.9% and lipids for 23.5 ± 4.9%. No statistically significant differences were observed (all *P* > .05). Total daily energy intake was also similar (2466 ± 231 vs 2577 ± 201 kcal, high vs low P-score, respectively), with protein intake corresponding to 1.34 and 1.38 g/kg/day when normalized to adjusted body weight.

### Renal and vascular parameters

Measured GFR, expressed either as absolute or BSA-indexed values, did not differ across tertiles. However, marked haemodynamic differences emerged: ERPF was significantly reduced in the middle and highest tertiles compared with the lowest (598 ± 45 and 505 ± 130 vs 756 ± 113 mL/min, *P* = .001), while FF progressively increased (26 ± 5% in the highest tertile vs 16 ± 3% in the lowest, *P* = .001) (Table 2). Consistently, renal vascular resistance was highest in the highest tertile compared with the lowest (0.11 ± 0.03 vs 0.07 ± 0.01 mmHg·min/mL, *P* = .012). No differences were observed in systemic haemodynamics, including central blood pressure, pulse pressure, augmentation index or cf-PWV.

**Table 2:** Renal haemodynamics and vascular parameters across P-score tertiles.

	First tertile	Second tertile	Third tertile	
	(low P-score)	(middle P-score)	(high P-score)	*P*-value
*n*	9	9	9	
P-score	–1.27 (–2.68 to –1.09)	–0.17 (–0.32 to –0.01)	1.49 (0.68–3.03)	**<.001**
mGFR (mL/min)	119 ± 20	110 ± 18	130 ± 39	.318
mGFR (mL/min/1.73 m^2^)	92 ± 15	85 ± 10	98 ± 21	.373
ERPF (mL/min)	756 ± 113**	598 ± 45	505 ± 130*	**.001**
FF (%)	16 ± 3	18 ± 2	26 ± 5*,**	**.001**
RVR (mmHg·min/mL)	0.07 ± 0.01	0.09 ± 0.01	0.11 ± 0.03*	**.012**
aSBP (mmHg)	111 ± 12	118 ± 16	120 ± 13	.440
aDBP(mmHg)	78 ± 12	79 ± 11	82 ± 10	.612
aMBP (mmHg)	89 ± 12	92 ± 12	95 ± 11	.550
PP (mmHg)	50 ± 9	47 ± 7	49 ± 11	.912
Aix@75 (%)	23.5 ± 11.6	25.1 ± 10.9	26.3 ± 12.3	.292
cf-PWV (m/s)	7.94 ± 1.17	8.07 ± 1.31	8.51 ± 1.10	.408
Brachial artery diameter (mm)	4.24 ± 0.47	4.08 ± 0.45	4.57 ± 0.81	.289
FMD (%), traditional	6.21 ± 1.76	5.11 ± 2.32	3.44 ± 1.49*	**.014**
FMD (%), allometric scaling	5.93 ± 1.69	4.96 ± 1.93	3.67 ± 1.51*	**.036**
Response to GTN (%)	6.80 ± 1.91	6.71 ± 1.89	7.01 ± 2.12	.412

Values are mean ± standard deviation or median (interquartile range).

*P*-values refer to overall comparisons across tertiles.

*P < .05 vs first tertile (low P-score).

**P < .05 vs second tertile (high P-score).

Aix, augmentation index.

Bold values indicate *P* < .05

Endothelial function showed a graded association with the P-score. Traditional FMD was significantly lower in the highest tertile compared with the lowest (3.44 ± 1.49 vs 6.21 ± 1.76%, *P* = .014), and similar results were obtained when accounting for arterial diameter (allometric scaling FMD, *P* = .036).

By contrast, endothelium-independent vasodilation in response to GTN did not differ across tertiles (*P* = .412) (Table 2).

### Relationship between the P-score, insulin resistance and vascular function

**Figure 1:**
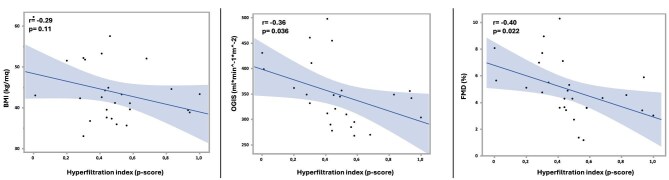
Associations of BMI, insulin sensitivity (OGIS) and FMD with the haemodynamic P-score. Scatter plots showing the relationship of BMI (**a**), OGIS (**b**) and FMD (**c**) with with the P-score.

In univariate correlation analyses, the P-score did not correlate with BMI (r = –0.29, *P* = .11) (Fig. 1a). Conversely, higher P-score values were associated with lower insulin sensitivity, as reflected by OGIS (r = –0.36, *P* = .036) (Fig. 1b), and with impaired endothelial function, as indicated by lower FMD (r = –0.40, *P* = .022) (Fig. 1c). The association with endothelial function remained significant when FMD was analysed using the allometric scaling approach (*P* = .028, R^2^ = 0.24).

**Figure 2:**
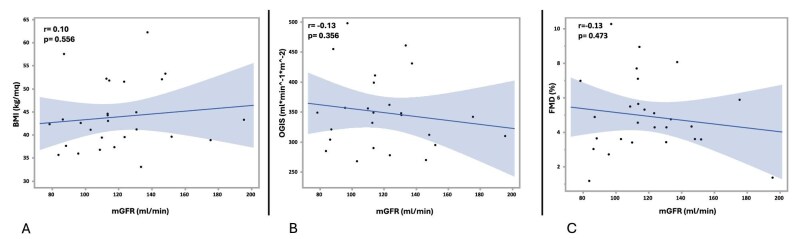
Associations of BMI, insulin sensitivity (OGIS) and FMD with mGFR. Scatter plots showing the relationship of BMI (**a**), OGIS (**b**) and FMD (**c**) with mGFR.

mGFR, instead, showed no association with BMI, OGIS or FMD, highlighting that mGFR alone does not capture the maladaptive component of hyperfiltration (Fig. 2).

Regarding arterial stiffness, neither the P-score nor mGFR showed significant associations with cf-PWV or Aix@75. Furthermore, both P-score and mGFR were not correlated with age. With respect to sex, P-score values did not differ between men and women, whereas mGFR tended to be higher in men than in women (135 ± 30 vs 113 ± 25 mL/min, *P* = .059).

To assess whether the significant associations observed in univariate analysis were independent of age and sex, multivariable analyses were performed. We constructed two models with OGIS as the dependent variable. Model A included P-score, BMI and age, while Model B included P-score, BMI and sex to avoid the overfitting. In both models, the P-score remained significantly associated with lower OGIS (Model A: st.β = –0.44, *P* = .032; Model B: β = –0.49, *P* = .017).

The association between P-score and OGIS remained significant in permutation testing (10 000 iterations, empirical *P* = .039).

Finally, we examined whether the association between P-score and FMD was independent of demographic factors. In multivariable models with allometrically scaled FMD as the dependent variable, adjusted for age and sex, the inverse association between P-score and FMD observed in univariate analysis was attenuated (st.β = –0.36, *P* = .062). Additional adjustment for BMI did not materially change the results.

### Sensitivity analysis

Excluding the four participants with diabetes (*n* = 23), the inverse associations of P-score with OGIS (r = –0.37, *P* = .047) and with allometrically scaled FMD (r = –0.39, *P* = .031) remained significant. In multivariable models, specified as in the main analysis, the association with FMD was attenuated after adjustment for age and sex (st.β = –0.40, *P* = .058), whereas the association with OGIS remained robust (st.β ≈ –0.5, *P* < .05) for both models (A and B).

## DISCUSSION

In this study, we introduced a haemodynamic index (P-score) to characterize the heterogeneity of glomerular hyperfiltration in severe obesity. The principal findings are that: (i) higher P-score values, reflecting a maladaptive phenotype with disproportionate elevation of FF relative to renal plasma flow, were consistently associated with insulin resistance as assessed by OGIS; (ii) P-score was inversely associated with endothelial function, as measured by FMD, an association that persisted when applying the allometric scaling approach; (iii) mGFR alone did not show these associations, underscoring the limits of absolute GFR and supporting the need for integrated haemodynamic assessment in obesity.

The observation that obesity is accompanied by renal hyperfiltration has been established for decades. In a seminal study, Chagnac and colleagues reported that severely obese, otherwise healthy adults had a 61% increase in mGFR and about 30% increase in effective renal plasma flow compared with lean controls, resulting in an elevated FF and higher albumin excretion despite the absence of overt kidney disease [5]. Similarly, in the early stages of type 2 diabetes, supraphysiological GFR has been reported in up to 40% of subjects [23].

Insulin resistance has consistently emerged as a key determinant of altered renal haemodynamics. Dengel *et al*. showed in obese, hypertensive adults that reduced clamp-measured insulin sensitivity correlated with higher FF, independent of body size. Even without absolute hyperfiltration by mGFR, the most insulin-resistant individuals displayed a profile consistent with maladaptive glomerular hypertension [24]. More recently, van Bommel *et al*. reported in adults with type 2 diabetes on metformin that insulin resistance correlated inversely with GFR and FF, independently of confounders [25].

In line with this, our pilot study shows that an exploratory index combining FF and ERPF correlated strongly with OGIS, supporting the view that maladaptive hyperfiltration reflects complex metabolic abnormalities beyond BMI.

Although the exploratory nature of the P-score needs confirmation in larger and independent cohorts, it is particularly relevant in our severely obese cohort, where uniformly high BMI failed to discriminate renal haemodynamic profiles. In this context, insulin resistance is a more informative determinant: despite similar adiposity, lower OGIS identified patients with a maladaptive phenotype, consistent with links to hyperinsulinemia. The present results align with our previous work in severely obese individuals without diabetes, where hyperfiltration affected about one-third and was linked to insulin resistance assessed by OGIS rather than BMI. In that study, OGIS correlated inversely with eGFR, and hyperfiltration normalized after bariatric surgery only in those who improved insulin sensitivity [26]. In a separate prospective cohort, we further observed that improvements in renal function after bariatric surgery were predicted by changes in renal vascular indices, particularly the resistive index, rather than by weight loss [21]. Taken together, these findings suggest that vascular and metabolic alterations, more than body size, drive renal outcomes in obesity.

Within this framework, endothelial dysfunction emerges as a critical consequence of insulin resistance in obesity, driven by hyperinsulinemia, reduced nitric oxide bioavailability and oxidative stress [27, 28]. Seminal work by Steinberg *et al.* [15] demonstrated that obese insulin-resistant individuals exhibited markedly blunted endothelium-dependent vasodilation despite preserved responses to endothelium-independent stimuli, directly linking impaired insulin signalling to reduced nitric oxide–mediated vascular relaxation.

Importantly, therapeutic interventions that improve insulin sensitivity also normalize renal haemodynamics. Pistrosch *et al.* demonstrated that treatment with rosiglitazone reduced GFR and FF in hyperfiltering type 2 diabetes subjects and lowered albuminuria by ∼65%, effects attributed to improved intrarenal nitric oxide activity [29]. Similarly, systemic microvascular function assessed by skin capillary recruitment, was directly related to mGFR and ERPF, and inversely to renal vascular resistance in overweight subjects with type 2 diabetes [30]. These studies emphasize that renal and systemic endothelial function are tightly interconnected in the context of overt diabetes. Notably, even in our largely nondiabetic obese cohort, P-score correlated inversely with FMD, indicating that the coupling between maladaptive renal haemodynamics and endothelial dysfunction can already be detected in obesity before overt diabetes, pointing to insulin resistance as the most probable driver.

Furthermore, our finding that higher FF fraction despite relatively preserved ERPF were inversely associated with FMD is consistent with the hypothesis that maladaptive renal haemodynamics mirror systemic endothelial dysfunction. Although adjustment for age attenuated this association, the relationship remained close to significance. This attenuation is likely explained by the strong inverse correlation between FMD and age, a well-recognized phenomenon that was also evident in our cohort. Given the limited sample size, collinearity between age and FMD may have reduced statistical power, but the persistence of trend (*P* = .062) suggests that renal haemodynamic stress and endothelial dysfunction remain closely interconnected.

In the light of these observations, renal hyperfiltration should not be regarded as a uniform condition.

A maladaptive phenotype shows elevated FF with reduced ERPF, reflecting intraglomerular hypertension, whereas a flow-driven pattern arises when higher ERPF sustains increased GFR without altering FF [31]. Our exploratory index (P-score) was designed to capture this distinction and clustered with metabolic and vascular alterations. This aligns with evidence that glomerular hypertension, rather than high flow, drives progressive kidney injury.

Several mechanisms have been proposed to drive glomerular hyperfiltration in obesity, including salt retention, activation of the renin–angiotensin–aldosterone and sympathetic nervous systems, and alterations in leptin signalling [32, 33]. While our study did not assess these pathways directly, the haemodynamic index we propose captures a profile compatible with intraglomerular hypertension. In this context, recent work has distinguished ‘absolute’ hyperfiltration, reflecting increased single-nephron GFR in conditions such as obesity, from relative compensatory hyperfiltration after nephron loss [32, 34]. This conceptual distinction emphasizes that the haemodynamic burden observed in obesity differs from, but ultimately converges with, the adaptive responses seen in chronic kidney disease.

Inflammation has also been implicated in obesity-related renal dysfunction [26]. In our cohort, however, hs-CRP levels did not differ across P-score groups, suggesting that baseline inflammatory status was unlikely to account for the haemodynamic differences observed.

Studies in remnant kidney models first established that hyperfiltration per nephron accelerates glomerular damage via increased capillary pressure [35, 36], a concept formalized as the ‘hyperfiltration theory’ [2]. Subsequent mechanistic work demonstrated that podocytes exposed to biomechanical overload detach and scar [37], while shear stress activates COX-2/PGE2 signalling [38], collectively underscoring FF as a more meaningful marker of maladaptive hyperfiltration than absolute GFR. We also considered dietary intake, as macronutrient composition can influence renal haemodynamics. Analysis of food diaries revealed no significant differences in the relative contributions of proteins, carbohydrates or lipids between groups. These findings argue against short-term macronutrient composition as an explanation for the haemodynamic differences we observed, although we cannot exclude the impact of long-term dietary patterns or sodium intake.

This study has limitations to address. Firstly, the sample size was relatively small, limiting the ability to include multiple confounders in regression models, and the cross-sectional design precludes causal inference. Secondly, the haemodynamic index was derived and standardized within this cohort and requires external validation. Finally, P-score was not designed to disentangle tubulo-glomerular feedback from tubular responses to pressure-mediated hyperfiltration. We did not assess specific tubular injury mechanisms; however, as none of the patients had proteinuria on urinalysis, overt tubular damage was unlikely.

Despite these limitations, our study is strengthened by detailed phenotyping, with gold-standard assessments of renal haemodynamics, vascular function and insulin sensitivity. This comprehensive approach revealed pathophysiological links that larger but less deeply profiled cohorts might miss.

In conclusion, by integrating FF and ERPF into a novel haemodynamic index, we provide evidence that insulin resistance is a key determinant of pressure-driven hyperfiltration dysfunction in severe obesity. These findings support the use of haemodynamic characterization beyond absolute GFR to better capture the renal consequences of obesity and its metabolic complications.

## Supplementary Material

sfaf334_Supplemental_Files

## Data Availability

Data that support the findings of this study are available from the corresponding author upon reasonable request.
