# Supplementary material for: Severe obesity associates with maladaptive glomerular haemodynamics clustered with insulin resistance and endothelial dysfunction
Source: Clin Kidney J. 2025 Nov 3;18(11):sfaf334. doi: 10.1093/ckj/sfaf334 (PMC12635466; doi:10.1093/ckj/sfaf334)
Supplement: sfaf334_Supplemental_Files [file sfaf334_supplemental_files.zip › Supplementary methods.docx]

***Supplementary methods***

*Arterial tonometry*

Aortic stiffness was assessed by carotid-femoral pulse wave velocity (cf-PWV). Waveforms were recorded at the femoral and carotid site, using SphygmoCor (AtCor Medical, Sydney, NSW, Australia). PWV was calculated from the pulse transit time using 80% of the direct distance method with a rigid sliding caliper, according to the formula: PWV= 0,8*d/TT, with d the surface distance between the carotid and the femoral site of measurement. Central hemodynamics was assessed by acquisition of peripheral pressure waveforms of the radial artery at the wrist, using applanation tonometry: a generalized validated transfer function (SphygmoCor software) was used to generate the corresponding central aortic pressures. Arterial waveforms were calibrated with brachial mean blood pressure, calculated as diastolic blood pressure + 0.4 * pulse pressure. Augmentation Index (Aix@75) was calculated as the ratio of augmentation pressure, the difference between the second and first systolic peak, and pulse pressure, expressed as a percentage and adjusted for heart rate of 75 bpm. An in-device quality rating of ≥80% was required for all recordings. All measurements were performed by trained staff with specific experience in obese individuals, following current international guidelines.

*Renal functional measurements*

All studies were performed after an overnight fasting (12h). Two antecubital veins were cannulated, one for tracer administration and one for blood sampling. Glomerular filtration rate was assessed by plasma clearance of iohexol. Five milliliters of iohexol (Omnipaque, 300 mg/mL; Nycomed, Milan, Italy) were injected over 30 seconds and immediately flushed with 10 mL of saline. Venous blood samples were collected at 5, 15, 60, 90, 120, 150, 180, 210 and 240 min after injection. Plasma concentrations of iohexol were determined by high-performance liquid chromatography (Perkin Elmer, Waltham, MA, USA). As expected, two chromatographic peaks corresponding to different isomeric forms were detected; the first was excluded. Peak heights were compared with a calibration curve to derive plasma concentrations. The decay curve of plasma iohexol was analyzed by a two-compartment model (DIMSUM discrimination model) to ensure precision in the estimate of GFR. The coefficients (A1, A2) and slopes (α1, α2) obtained from the biexponential fit were used to calculate the area under the curve (AUC). Iohexol clearance was then computed as the injected dose (double-weighed syringe) divided by AUC. GFR was expressed as absolute values and after adjustment for body surface area (BSA) or for height [18].

Effective renal plasma flow (ERPF) was measured using the multiple-sample plasma clearance of ^123^I-ortho-iodohippurate (^123^I-OIH; Covidien, Dublin, Ireland). A bolus of approximately 7 MBq of tracer was administered intravenously. Venous blood samples were collected at 5, 10, 15, 20, 30, 40, and 60 minutes, centrifuged, and plasma aliquots were counted in a gamma counter (Atomlab 950; Biodex, Shirley, NY, USA). Injected dose was obtained by multiplying the final value of the standard corrected value for the dilution factor by the ratio between activity (in megabecquerels) of the injected dose and standard [19]. Multiple blood sample clearance curves were fitted with a two-exponential model using the curve-fitting tool of the software MatLab 7.9.0 (The Mathworks, Natick, Massachusetts), and RPF was calculated as follows: RPF (milliliters per minute) = injected dose / (A / α + B / β), in which A and B are the y-axis intercepts of each exponential component and α and β are the respective slopes [18]. RPF was expressed as an unadjusted value, as well as being adjusted for BSA and for height. Filtration fraction (FF) was computed as the ratio between mGFR and ERPF. Because FF is dimensionless, it is not affected by the method of indexing and is considered a robust descriptor of renal haemodynamics in obesity. Renal vascular resistance (RVR) was calculated as mean arterial pressure divided by renal blood flow, the latter derived as ERPF/(1 − haematocrit).

*Oral Glucose Insulin Sensitivity (OGIS)*

Insulin sensitivity was assessed by the oral glucose insulin sensitivity (OGIS) index, calculated from plasma glucose and insulin concentrations obtained during a standard 75-g OGTT at 0, 120, and 180 minutes. The computation follows the mathematical model developed by Mari et al. [17], which uses fixed parameters optimized against data from euglycemic–hyper insulinemic clamps.

The method describes glucose kinetics as: dG/dt= -(S_G_ + X(t))*G(t) + S_G_*G_b_, where:

- G(t) is plasma glucose concentration,

-Gb is basal glucose

-S_G_ is glucose effectiveness

X(t) is insulin action (driven by plasma insulin through a dynamic transfer function).

From this system a steady-state glucose clearance is derived. The model includes six constants calibrated against clamp data.

OGIS provides an estimate of glucose clearance (ml·min⁻¹·m⁻²) that would correspond to clamp conditions. Lower values indicate reduced insulin sensitivity, while higher values reflect preserved insulin action. The index has been validated in lean, obese, and type 2 diabetic subjects, and correlates strongly with clamp-derived insulin sensitivity. It has also been shown to track longitudinal changes after lifestyle or surgical interventions.
