# Supplementary material for: Severe obesity associates with maladaptive glomerular haemodynamics clustered with insulin resistance and endothelial dysfunction
Source: Clin Kidney J. 2025 Nov 3;18(11):sfaf334. doi: 10.1093/ckj/sfaf334 (PMC12635466; doi:10.1093/ckj/sfaf334)
Supplement: sfaf334_Supplemental_Files [file sfaf334_supplemental_files.zip › supplementary s1 table.docx]

|  | **Total cohort** |
| --- | --- |
| n | 27 |
| Age (years) | 46 ± 12 |
| Sex, F(%) | 19 (70) |
| Weight (kg) | 121 ± 21 |
| BMI (kg/m^2^) | 43.9 ± 7.2 |
| Smoking, n (%) | 5 (19) |
| Hypertension, n (%) | 8 (30) |
| Dyslipidemia, n (%) | 10 (39) |
| Newly onset T2D, n(%) | 4 (15) |
| SBP (mmHg) | 128 ± 13 |
| DBP (mmHg) | 79 ± 9 |
| Heart Rate (bpm) | 71 ± 10 |
| eGFR (ml/min/1.73m^2^) | 99 ± 16 |
| eGFR (ml/min) | 129 ± 25 |
| mGFR (ml/min/1.73m^2^) | 91 ± 26 |
| mGFR (ml/min) | 121 ± 20 |
| Glycaemia (mg/dl) | 120 ± 21 |
| HbA1c (%) | 6.1 ± 0.7 |
| Total Cholesterol (mg/dl) | 183 ± 24 |
| HDL (mg/dl) | 42 ± 11 |
| LDL (mg/dl) | 120 ± 21 |
| Triglycerides (mg/dl) | 116 [89-151] |

**Supplementary Table 1.**
